# Vertebral Body Morphology in Neuromuscular Scoliosis with Spastic Quadriplegic Cerebral Palsy

**DOI:** 10.3390/jcm13206289

**Published:** 2024-10-21

**Authors:** Göker Utku Değer, Heon Jung Park, Kyeong-Hyeon Park, Hoon Park, Mohammed Salman Alhassan, Hyun Woo Kim, Kun-Bo Park

**Affiliations:** 1 Department of Orthopedics and Traumatology, Beykoz State Hospital, Istanbul 34800, Türkiye; gokerdeger@hotmail.com; 2 Division of Pediatric Orthopedic Surgery, Severance Children’s Hospital, Yonsei University College of Medicine, Seoul 03722, Republic of Korea; ryan0912@naver.com (H.J.P.); ospkh@yuhs.ac (K.-H.P.); drmsalhassan@gmail.com (M.S.A.); pedhkim@yuhs.ac (H.W.K.); 3 Department of Orthopedic Surgery, Gangnam Severance Hospital, Yonsei University College of Medicine, Seoul 06273, Republic of Korea; hoondeng@yuhs.ac

**Keywords:** pedicle, vertebral body, neuromuscular scoliosis, idiopathic scoliosis, cerebral palsy

## Abstract

**Background/Objectives**: The distorted vertebral body has been studied in scoliosis; however, there is little knowledge about the difference between neuromuscular and idiopathic scoliosis. This study aimed to investigate the vertebral body morphology in patients with spastic quadriplegic cerebral palsy and scoliosis (CP scoliosis) and compare them with those of apex- and Cobb angle-matched patients with adolescent idiopathic scoliosis (AIS). **Methods**: Thirty-four patients with CP scoliosis and thirty-two patients with AIS were included. The pedicle diameter, chord length, and vertebral body rotation were evaluated at one level above the apex, one level below the apex, and at the apex using a reconstructed computed tomography scan. The apex of the curve and Cobb angle were too diverse between patients with CP scoliosis or AIS. Eighteen patients were matched in each group according to the apex and Cobb angle (within 5-degree differences) of the major curve, and compared between matched groups (mCPscoliosis vs. mAIS). **Results**: In the comparison of the apex and Cobb angle-matched groups, there was no statistical difference in the Cobb angle between mCPscoliosis (80.7 ± 13.8 degrees) and mAIS (78.6 ± 13.6 degrees, *p* = 0.426), and the vertebral body rotation (25.4 ± 15.4° in mCPscoliosis vs. 24.4 ± 6.5° in mAIS, *p* = 0.594). There was no difference in the pedicle diameters of either the convex (3.6 ± 1.1 mm in mCPscoliosis vs. 3.3 ± 1.2 mm in mAIS, *p* = 0.24) or concave side (3.1 ± 1.2 mm in mCPscoliosis vs. 2.7 ± 1.6 mm in mAIS, *p* = 0.127). However, the patients in the mCPscoliosis group were younger (12.7 ± 2.5 years vs. 14.6 ± 2.4 years, *p* = 0.001), and the chord length was shorter on the convex (38.0 ± 5.0 mm vs. 40.4 ± 4.9 mm, *p* = 0.025) and concave (37.7 ± 5.2 mm vs. 40.3 ± 4.7 mm, *p* = 0.014) sides compared with those of the mAIS group. **Conclusions**: With a similar apex and Cobb angle, the vertebral body rotation and pedicle diameter in patients with CP scoliosis were comparable to those with AIS; however, the chord length was shorter in CP scoliosis. For the selection of the pedicle screw in CP scoliosis, the length of the pedicle screw should be more considered than the diameter.

## 1. Introduction

Scoliosis is defined as a coronal plane deformity of more than 10 degrees in the spine; however, it is a three-dimensional deformity that includes vertebral body rotation (VBR). A pedicle screw is inserted during scoliosis correction to fix all three columns and achieve sufficient correction through rod derotation, distraction, compression, and direct vertebral body rotation. Pedicle morphology must be clarified to insert the pedicle screws correctly and avoid complications. Many previous studies investigating idiopathic scoliosis involving an examination of altered pedicle morphology and the determination of the safe limits for pedicle screw applications have been performed [1,2,3]. In these reports, the concave-side pedicle diameter (PD) was narrower, and VBR was severe around the curved apex [4,5]. It is believed that the conditions in neuromuscular scoliosis may be similar, but the differences or similarities between neuromuscular and idiopathic scoliosis have not yet been comprehensively studied.

The Cobb angle at the time of diagnosis or operation is usually larger for neuromuscular scoliosis, and progression is more rapid than for idiopathic scoliosis. A greater-than-40-degree Cobb angle was detected in almost 40% of cerebral palsy patients (GMFCS III–V) at a mean age of 11 years, and the prevalence reached 62% in patients with non-ambulatory cerebral palsy (CP) [6]. Additionally, the highest complication rates after scoliosis surgery in patients with CP were reported, reaching up to 17% [7,8]. There are many reasons for the high complication rate, such as poor oral intake, seizure, cardiopulmonary problems, and severe curves. One of the important factors related to the complications in scoliosis with CP may be the severely distorted vertebral body at high degrees of Cobb angle. It is even more important to understand the severely distorted pedicle morphology in scoliosis with CP because scoliosis correction techniques have evolved with advances in pedicle screw instrumentation [9,10,11,12].

Several previous studies examined the vertebral morphology of neuromuscular scoliosis cases only in diverse disease groups with neurofibromatosis, spinal muscular atrophy, Chiari malformation, or a small number of mixed diagnosed groups [13,14,15,16,17]. No comprehensive study has yet examined pedicle morphology in only patients with spastic quadriplegic cerebral palsy and scoliosis. Furthermore, there have been few reports about the comparison of vertebral body morphology between the different disease entities. In the present study, we selected only spastic quadriplegic cerebral palsy scoliosis (CP scoliosis) and compared the altered vertebral morphology with that of adolescent idiopathic scoliosis.

Among the various forms of neuromuscular scoliosis, patients with CP scoliosis are usually non-ambulatory, the curve magnitude is severe, and the onset is very young; hence, osteoporosis may be common, and the vertebral body would be skeletally immature. We hypothesized that the vertebral body rotation and pedicle morphology in CP scoliosis may differ from adolescent idiopathic scoliosis (AIS). However, the prerequisite for comparing different etiologies is that the curve character should at least be similar between groups. In the present study, we (1) evaluated VBR and pedicle morphology in non-ambulatory patients with CP scoliosis or patients with AIS and (2) compared those parameters between patient groups of CP scoliosis and AIS with apex and Cobb angle-matches of the major curve, using reconstructed computed tomography (CT) images.

## 2. Materials and Methods

### 2.1. Patient Selection

This retrospective study was approved by the Yonsei University Health System, Severance Hospital, Institutional Review Board (approval number: 4-2022-0937), and was conducted in accordance with the Declaration of Helsinki 2013. Informed consent waiver was obtained from the Institutional Review Board. The primary outcome of this study was to compare pedicle width between CP scoliosis and AIS. While there has not been a similar study, we found some pedicle diameter data in previous research [17]. The pedicle width was 4.7 ± 1.0 mm for CP and 4.2 ± 0.9 mm for AIS between T8 and L3, the common apex of scoliosis. Based on this data, we calculated a sample size of 33 with a type I error of 0.05 and a power of 0.9 for each group.

Among all patients who underwent surgery for scoliosis in our clinic between 2019 and 2022, the data of those with available reconstructed CT images for surgery planning were extracted. Of the 129 patients who met these criteria, 76 were found to have neuromuscular scoliosis, and 35 were diagnosed with idiopathic scoliosis. Eighteen patients with congenital or syndromic scoliosis were excluded.

**Figure 1:**
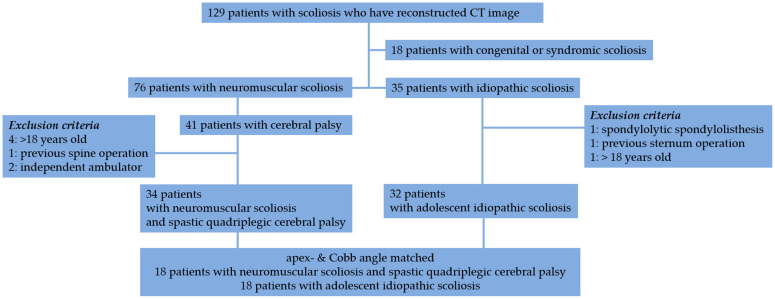
Flow diagram of the inclusion and exclusion process.

Forty-one patients were diagnosed with cerebral palsy and neuromuscular scoliosis. We excluded four patients who were older than 18 years, had undergone previous spine operations, or were independent ambulators. In 35 patients with idiopathic scoliosis, 3 patients were excluded because of spondylolytic spondylolisthesis, previous sternum operation, or being over 18 years old. Finally, 34 patients with spastic quadriplegic cerebral palsy and scoliosis (CP scoliosis) and 32 patients with adolescent idiopathic scoliosis (AIS) were enrolled in the study (Figure 1).

Many patients with AIS had smaller Cobb angles than those with CP scoliosis, and the apex in AIS was usually the middle of the thoracic spine. The apices of CP scoliosis were T8 (1), T9 (4), T10 (4), T11 (3), T12 (8), L1 (3), L2 (4), L3 (7), but those of AIS were T7 (3), T8 (4), T9 (7), T10 (5), T11 (2), T12 (4), L1 (2), L2 (4), and L3 (1). Because the vertebral body between thoracic and lumbar is different, the comparison of the vertebral body morphology should be performed at the same level. Additionally, the severity of Cobb angle may affect to the vertebral body morphology. We collected patients with same apex of the major curve and a similar major Cobb angle within 5-degree differences in both groups.

Comparison to find the difference in vertebral body morphology between CP scoliosis and AIS was performed using data for each of the 18 patients with CP scoliosis (mCPscoliosis) and AIS (mAIS) matched for apex and Cobb angle (within 5-degree differences) of the major curve. The matched apices were T8 (1), T9 (2), T10 (4), T11 (2), T12 (3), L1 (2), L2 (3), and L3 (1).

### 2.2. Radiographic Measurements and Reconstruction of CT Images

All patients had a whole spine posteroanterior and lateral view radiographs taken in the preoperative evaluation. Cobb angle measurements were taken from whole spine posteroanterior radiographs obtained in the sitting position for CP scoliosis and standing position for AIS using the line parallel to the upper endplate of the uppermost end vertebra and the lower endplate of the lowermost end vertebra. The curve’s apex was defined as where the curvature is most pronounced or severe. If the disc was the apex in CP scoliosis, the above or below vertebra of AIS was defined as the apex for the matching.

CT scans were performed from the T1 to the S1 vertebrae with the patient in the supine position using SOMATOM (Siemens Healthineers, Erlangen, Germany). The resulting CT images were reconstructed using Syngo.via VB40 software (Siemens Healthineers, Erlangen, Germany). The image contrast levels were standardized for clear soft tissue and bone demarcation at the vertebral pedicles. The relevant vertebral body was identified by counting upward from the sacrum and confirmed by counting the rib levels superiorly and inferiorly. From the reconstructed sagittal and coronal images, a transverse image parallel to the endplate plane in both the sagittal and coronal plane was reconstructed at the center of the pedicle. When the superior and inferior endplate planes were not parallel owing to vertebral wedging, an orientation approximately halfway between (i.e., bisecting) the two endplate inclinations was selected [18,19].

**Figure 2:**
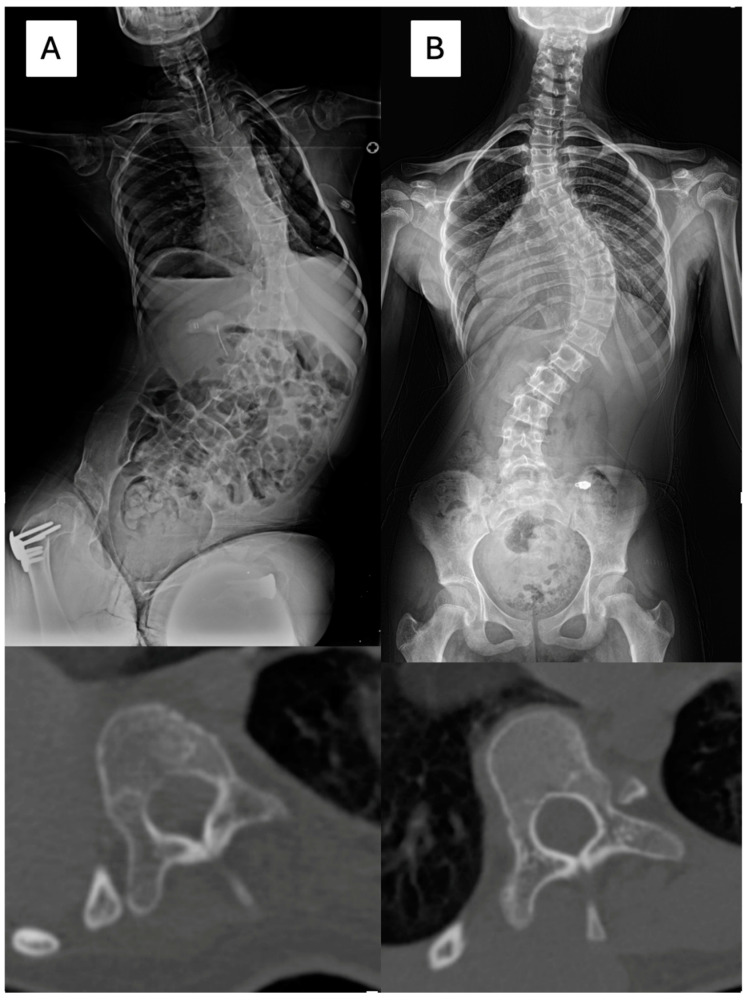
(**A**) A female aged 10 years and 7 months had neuromuscular scoliosis with spastic quadriplegic cerebral palsy. The Cobb angle between T6 and L4 was 79 degrees, and the apex was T10 (CT). The pedicle diameter and chord length were 3.2 and 24.9 mm at the concave side, and 4.8 and 29 mm at the convex side. (**B**) An 11 years, 6 months old female was diagnosed with adolescent idiopathic scoliosis with a Cobb angle of 74 degrees (T7-L1) and apex at T10 (CT). The pedicle diameter and chord length were 3.9 and 37.5 mm at the concave side, and 4.8 and 39 mm at the convex side.

**Figure 3:**
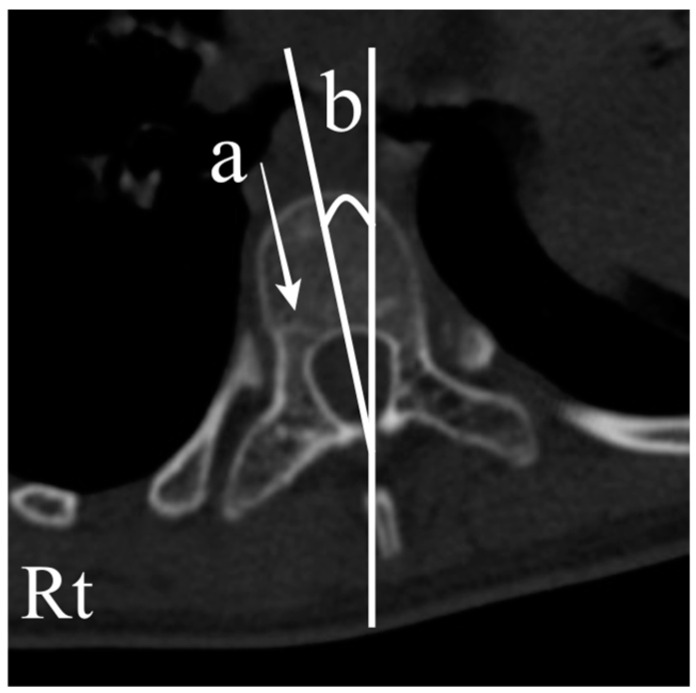
Exemplar vertebra with neurocentral synchondrosis (a) and a vertebral body rotation toward the right side (b).

In total, 102 vertebrae with CP scoliosis and 96 vertebrae with AIS were measured. Pedicle diameter (PD), chord length (CL), and vertebral body rotation (VBR) were evaluated one level above the apex, one level below the apex, and at the apex using a reconstructed CT scan [14,17,18,19]. PD was measured in the isthmus region, where the medial and lateral middle cortical borders were the narrowest. The CL was measured as the distance between the posterior cortical entry point of the pedicle and the anterior vertebral cortex in line with the axis of the pedicle. VBR was measured as the angle between the vertical line and the line that bisects the vertebral body (Figure 2). Additionally, the presence of neurocentral synchondroses was investigated (Figure 3) [20,21].

### 2.3. Statistical Analyses

All statistical analyses were performed using SPSS version 25.0 (IBM Corp., Armonk, NY, USA), and *p* < 0.05 was considered statistically significant. Data were assessed for normality using the Shapiro–Wilk test. Paired *t*-test or independent *t*-tests were used to compare continuous variables between the convex and concave sides of the CP scoliosis or AIS, and between VBR, PD, and CL of the apex- and Cobb angle-matched groups (mCPscoliosis vs. mAIS). The chi-square test and Fisher’s exact test were used to compare categorical variables according to expected values. Among the radiographic parameters, VBR, PD, and CL at the apex of matched groups were measured again by experienced orthopedic surgeons to estimate interobserver reliability. The intraclass correlation coefficient to define interobserver reliability was calculated as 0.982 (0.964–0.991) for VBR, 0.910 (0.857–0.944) for PD, and 0.777 (0.645–0.860) for CL.

## 3. Results

### 3.1. Patient Characteristics

The mean age at the time of surgery for CP scoliosis was 14 years and 2 months (range, 10–18 years). Fifteen patients were male, and 19 patients were female. The mean height and weight were 138.3 ± 14.5 cm (108–173 cm) and 30.1 ± 11.5 kg (15–62 kg), respectively. Thirteen patients were classified as Gross Motor Function Classification System (GMFCS) IV and 21 patients as GMFCS V. Twenty-one patients had dislocated hips and 19 patients had undergone reconstruction operations. The mean age of the AIS patients was 14 years and 7 months (range, 11–18 years). One patient was male and 31 patients were female. The mean height and weight were 154.5 ± 8.95 cm (127–170 cm) and 46.3 ± 9.8 kg (28–83 kg), respectively.

### 3.2. Comparison Between the Convex and Concave Side in Patients with CP Scoliosis

The average Cobb angle of the main curve was 87.7 ± 18.7°, and T12 was the most common apex of the curve. Neurocentral synchondrosis was noted in 55.8% (19/34) of the patients. Left thoracic curve was noted in 32.4% (11/34) of patients, and the average VBR was 29.9 ± 14.6°. The PD of the convex side (4.0 ± 1.5 mm) was larger than that of the concave side (3.6 ± 1.4 mm, *p* = 0.001). The chord length (CL) of the convex side (39.2 ± 5.0 mm) and concave side (39.0 ± 5.5 mm) showed no statistically significant differences (*p* = 0.550).

### 3.3. Comparison Between the Convex and Concave Side in Patients with AIS

The average Cobb angle of the main curve was 68.2 ± 18.8°, and T9 was the most common apex of the curve. Neurocentral synchondrosis was noted in 6.3% (2/32) of the patients. All patients had a right thoracic curve, and the average VBR was 18.7 ± 9.7°. The PD of the convex side (3.4 ± 1.2 mm) was larger than that of the concave side (2.6 ± 1.5 mm, *p* < 0.001). The chord length (CL) of the convex side (38.6 ± 5.9 mm) and concave side (39.4 ± 5.6 mm) showed no statistically significant differences (*p* = 0.414).

### 3.4. Comparison of Apex- and Cobb Angle-Matched Patients with CP Scoliosis and AIS

The average Cobb angles were 80.7 ± 13.8 degrees and 78.6 ± 13.6 degrees in the matched CP scoliosis (mCPscoliosis) and AIS (mAIS) groups (*p* = 0.426). The average age was younger in the mCPscoliosis (12.7 ± 2.5 years) than the mAIS (14.6 ± 2.4 years, *p* = 0.001). In mCPscoliosis, males were predominant, while the height and weight were greater in m AIS.

There was no difference between the two groups in terms of VBR (25.4 ± 15.4° in mCPscoliosis vs. 24.4 ± 6.5° in mAIS, *p* = 0.594) or PD in either the convex (3.6 ± 1.1 mm in mCPscoliosis vs. 3.3 ± 1.2 mm in mAIS, p=0.24) or concave side (3.1 ± 1.2 mm in mCPscoliosis vs. 2.7 ± 1.6 mm in mAIS, *p* = 0.127). However, the CL of the mCPscoliosis was significantly shorter than the mAIS in both the convex side (38.0 ± 5.0 mm in mCPscoliosis vs. 40.4 ± 4.9 mm in mAIS, *p* = 0.025) and concave side (37.7 ± 5.2 mm in mCPscoliosis vs. 40.3 ± 4.7 mm in mAIS, *p* = 0.014) (Table 1).

## 4. Discussion

Although it is commonly believed that scoliosis in cerebral palsy (CP) progresses with greater angular deformities and more pronounced differences in the vertebral structure compared to adolescent idiopathic scoliosis (AIS), our study revealed fewer differences than expected when we compared the CP scoliosis and AIS with matching apex and Cobb angle. While immature skeletal development is evident in both the younger average age of the CP scoliosis group and the higher prevalence of neurocentral synchondroses, it is noteworthy that, contrary to expectations, there is no significant difference in pedicle diameters and vertebral body rotation between the two matched groups. The only significant difference in vertebral structure is that cord length is smaller in the mCPscoliosis group compared with mAIS. These findings suggest that length, rather than diameter, should be prioritized when selecting implants in CP scoliosis.

Hong et al. reported 15 degrees of VBR with a Cobb angle of 60° in a diverse type of CP scoliosis [14], while Modi et al. reported 42 degrees of VBR with a Cobb angle of 74° [22]; however, only 6 of 24 patients were diagnosed with CP. In this study, the VBR of CP scoliosis was 29.9 degrees with Cobb angles of 87.7° in only patients with spastic quadriplegic cerebral palsy. In the matched groups, the VBR was 25.4 degrees with 80.7 Cobb angle. With an increase in the Cobb angle, VBR would increase, but there may be some differences according to the level of spasticity. However, the VBR increases with Cobb angle increase, regardless of the etiology. In AIS, the VBR was 18.7 degrees with Cobb angle of 68.2 degrees, and the VBR was 24.4 degrees with 78.6 Cobb angle.

Wang et al. found no statistical differences in PDs between the concave and convex sides, except at the apical area in neuromuscular scoliosis with Chiari malformation, but Chiari malformation is different from cerebral palsy [13]. Hong et al. reported that there were no significant differences between the concave and convex sides when the PD was compared in the 11 patients with CP; however, the Cobb angle was smaller than that in our study [17]. When we compared the PD in CP scoliosis, the convex side diameter was larger than that on the concave side like previous studies and it was similar in AIS groups. Although there may be differences according to the etiology of neuromuscular scoliosis or severity, the pedicle width would be larger on the convex side around the apex in patients with CP scoliosis.

In vertebral morphology studies investigating AIS, the pedicle diameter on the concave side was smaller than on the convex side, and the apical vertebra was the most rotated vertebra [4,17,18,23,24]. These findings on AIS were similar to our findings on the CP scoliosis group. Specifically, we found that patients with CP scoliosis were younger and the CL was shorter, but the PD was similar to that of the apex- and Cobb angle-matched patients with AIS. Although the etiology of AIS is uncertain, recent studies have reported the importance of neurocentral synchondrosis [20,21]. We suspected that there might be some differences in the presence of neurocentral synchondrosis and growth patterns between AIS and CP scoliosis. Neurocentral synchondrosis was commonly noted in patients with CP scoliosis compared with AIS and the patients with CP scoliosis were younger in the matched comparison. The vertebral body was immature in CP scoliosis, which may be related to the shorter CL. However, there was no difference in the PD between the groups. One of the reasons for this may be the thin cortical bone in CP scoliosis, due to the high levels of osteoporosis. Hell et al. found significantly smaller vertebral body and PDs in neuromuscular scoliosis with spinal muscular atrophy as compared to age-matched healthy controls [15], which may be related to the early onset of neuromuscular scoliosis and the long-standing adverse effects on the neurocentral synchondroses. We think that the PD in CP scoliosis may be narrower with aging than those in AIS because of earlier suppression of neurocentral synchondrosis. In CP scoliosis, CL was short due to skeletal immaturity, but the PD may not be so small because of thin cortical bone due to osteoporosis when compared with similar Cobb angle AIS. With growth, the PD would be smaller with the long-standing adverse effect on neurocentral synchondrosis, so when we operate on CP scoliosis in older patients, the PD looks very small. Future studies should be conducted to investigate the severity of osteoporosis and the effect of neurocentral synchondroses.

Several studies have reported a smaller PD in neuromuscular scoliosis, resulting in the use of hooks or wiring. Sarwahi et al. compared the pedicle morphology between different etiology like our study, although they compared neurofibromatosis type1 patients with AIS [5]. They found a higher incidence of abnormal pedicle and higher misplacement of pedicle screws compared to AIS. Abnormal pedicles were found in 69% of the scoliosis cases diagnosed with neurofibromatosis, and they include cases with cancellous pedicle diameter less than 4 mm, according to the classification developed by Sarwahi et al. [5] and revised by Li et al. [14]. However, the pedicle in neurofibromatosis is different from CP scoliosis. In this study, the PD in patients with CP scoliosis was not so small compared with those with apex- and Cobb angle-matched AIS. Instead, the results of our study suggest that a shorter pedicle screw length may be more important. The narrow PD in CP scoliosis would be related more to the larger Cobb angles with growth. For safe and correct pedicle screw placement, we recommend performing the operation in the early stages of CP scoliosis. Still, the correct pedicle screw placement in CP scoliosis is difficult. Because of the osteoporosis, the pedicle screw can easily break the inner or outer pedicle wall.

This study has several limitations which should be considered. The number of matched cases was relatively small because the Cobb angle was usually larger in patients with CP scoliosis, the apex was different (AIS has a relatively higher apex), and cases with CT images were small. Furthermore, we did not consider the curve pattern, such as a long C curve, a double curve, or a hip problem. However, we only selected spastic quadriplegic cerebral palsy of a single etiology and found a different vertebral morphology compared with apex- and Cobb angle-matched AIS using reconstructed CT images. In matching the groups, we used the standing radiography for AIS and sitting radiography for CP scoliosis because patients with CP suffered from spastic quadriplegia. There may be some differences between sitting and standing whole spine radiography. However, we do not take preoperative supine or prone radiography routinely for all patients. Later, supine or prone radiography may be used to measure the Cobb angle and matched analysis. Further studies that consider age and sex differences or supine and prone radiography should be conducted to address these limitations, and studies with larger populations should be followed to increase the power of the study.

## 5. Conclusions

The VBR in patients with CP scoliosis was comparable to that in patients with AIS matched for Cobb angle and apex. Although the patients were young and the vertebral body was smaller in terms of a short chord length in the patients with CP scoliosis, the pedicle diameters were similar between the two patient groups. These deformities may be related to osteoporosis caused by non-ambulation and immature vertebral body. In CP scoliosis, we should consider not only a smaller diameter but also a shorter length of pedicle screws. Pre-operative CT analyses of vertebra morphology is essential in CP scoliosis.

## Figures and Tables

**Table 1:** Comparison of vertebral body morphology between Cobb angle- and apex-matched patients with spastic quadriplegic cerebral palsy and scoliosis (mCPscoliosis) and adolescent patients with idiopathic scoliosis (mAIS).

	mCPscoliosis	mAIS	*p* Value
Cobb angle (degree)	80.7 ± 13.8	78.6 ± 13.6	0.426
Age * (years)	12.7 ± 2.5	14.6 ± 2.4	0.001
Sex (M:F)	7:11	1:17	<0.001
Height * (cm)	140.3 ± 16.5	152.9 ± 8.6	<0.001
Weight * (kg)	30.7 ± 13.2	43.1 ± 7.4	<0.001
Vertebral body rotation (degree)	25.4 ± 15.4	24.4 ± 6.5	0.594
Convex pedicle diameter (mm)	3.6 ± 1.1	3.3 ± 1.2	0.24
Convex chord length * (mm)	38 ± 5	40.4 ± 4.9	0.025
Concave pedicle diameter (mm)	3.1 ± 1.2	2.7 ± 1.6	0.127
Concave chord length * (mm)	37.7 ± 5.2	40.3 ± 4.7	0.014

Values are presented as mean ± standard deviation. *: *p* < 0.05.

## Data Availability

The datasets used and/or analyzed in the current study are available from the corresponding author on reasonable request.
